# Ghana health service performance appraisal system: a cross-sectional study on practices and perceptions among employees in the Bono East Region of Ghana, West Africa

**DOI:** 10.11604/pamj.2023.44.188.38581

**Published:** 2023-04-20

**Authors:** Mustapha Hallidu, Mubarick Nungbaso Asumah, Shadrach Asamoah-Atakorah, Fred Adomako-Boateng, Abukari Yakubu

**Affiliations:** 1 Department of Nursing and Midwifery, Kintampo Municipal Hospital, Ghana Health Service, Bono East, Ghana,; 2 Department of Global and International Health, School of Public Health, University for Development Studies, Tamale Northern Region, Ghana,; 3 Department of Physician Assistantship Medical, College of Health and Well-Being, Kintampo, Bono East, Ghana,; 4 Regional Health Directorate, Ghana Health Service, Kintampo, Bono East Region, Ghana,; 5 Human Resource Unit, Regional Health Directorate, Kintampo, Bono East Region, Ghana

**Keywords:** Appraisal, employees, evaluation, performance, practice

## Abstract

**Introduction:**

the fulcrum of every organization lies in the productivity of its employees. Consequently, organizations have adopted a policy of fostering an organizational culture that promotes employee development, resulting in the consistent use of performance evaluation systems particularly, performance appraisals (PA). Nonetheless, the issue of concern for several organizations is whether PA will truly be an adequate tool for maximizing employee´s performance. Here is where it becomes important to gauge the perceptions and practices of Ghana Health Service (GHS) employees in the Bono East Region of Ghana, West Africa, towards these performance appraisal systems (PAS).

**Methods:**

the study used an institutional-based cross-sectional study. Using simple random sampling, 375 health workers were recruited using a structured questionnaire. Data was analyzed using Stata. Bivariate and multivariate logistic regression analysis was performed and p-value less than 0.05 was considered statistical significance.

**Results:**

the study revealed that the majority of the respondents (86.7%) had completed the performance appraisal form. Out of which 47.7% complete and submit PA every year, followed by those who complete and submit during promotion time (24.3%), every half year (15.1%) and anytime (12.9%). The key determinants of PA completion include; increasing age (AOR=2.24, 95% CI: 1.08-4.62), male staff (AOR=2.38, 95% CI: 1.16-4.91), permanent GHS employees (AOR=2.9, 95% CI: 1.07-7.86), respondents who had worked for 3 to 7 years (AOR=5.53, 95% CI: 2.48-12.36) and those with over 7 years working experience (AOR=20.80, 95% CI: 5.43-79.74).

**Conclusion:**

the study identified that the majority of the respondents completed PA, but quite an encouraging number of them do not complete PA at the required period as expected. Age, male staff, permanent employment, and increasing years of experience were the significant predictors of PA completion. Continuous professional development for GHS staff on PAS is required. The GHS human resource division should organize stakeholders´ engagement with facilities managers and employees on the PAS to inform all managers and employees on what is required from everyone.

## Introduction

Performance appraisal (PA) is a recognized practice in institutions that is grounded in objective responsibilities with mental components, compared performance and project performance [1]. PA encompasses a lot of organizational practices, including measurement, objectives formulation, and reward management [1,2]. It further evaluates the achievements and limitations of employees and estimates if they require training or promotion going forward [1,3]. Performance Appraisal Systems (PAS) are usually utilized by both the government and private sectors. It includes assessing performance based on the verdicts and views of subordinates, colleagues, supervisors, managers, and even the employees themselves [2]. According to studies carried out across the globe, PAS is a ubiquitous means of assessing employees performance at workplaces either government, private or non-governmental institutions [4-7].

Performance Appraisal Systems (PAS) was inaugurated in the 1940s in the United States of America (USA) during the era of World War II. It was as a way of satisfactorily ascertaining employees´ remunerations then described as merit rating [8]. It was standardized around the 1950s as a prospective tool for managing employees rewards through pay increases out of which employees were counseled, demoted, and promoted or identified to be laid off [8,9]. Currently, PAS is among the cardinal components of any organization. According to Canet-Giner *et al*. [6], PA can have both administrative and development determinations; thus geared towards the fundamentals of decisions made on working conditions, promotions, dismissals, and rewards. Similarly, it can be employee development; where it focuses on advancing and developing employees´ capacities once their weaknesses and potential causes of those limitations are identified. That way, through effective communication of PA consequences, the organization can give feedback and proffer advice on effective work behaviors or design specific training plans [6].

In Ghana, PA was officially introduced into the civil service as part of a wider public sector reform in 1993 as a way of making the service more effective and efficient in achieving its mandates [10]. The Ghana Health Service (GHS) PAS was introduced with the objective of increasing the performance of its staff and again linking the individual and the institutional performance to realize critical health goals [10]. This process led to the construction of a new appraisal instrument which was piloted in Volta, Central, Eastern and Brong-Ahafo Regions of Ghana. This was followed by the training of managers and supervisors. Since the formation of management, the determination to advance performance has been a sacrosanct principle which eats into new areas daily [11]. The enhancement in the efficiency and effectiveness of employees as the very relevant institutional resources, is real through their PAS [12]. It is instructive to note that employees´ knowledge on PAS has a considerable positive relationship with performance and institutional commitment [13]. To make a PA a practical management tool from a broader perception, institutions and scholars spend time in training managers in PA skills, in developing system assessment which factor in reliability, validity and managerial aims as developing systems to actualize specific institutional conditions and aspirations.

In light of the ascribed significance of PAS and the objective of achieving its effectiveness, several studies have revealed that to realize an effective PAS, giving recognition to the perceptions of the objectivity of the PAS and their feedback to this system are as needful as the scores issued by the appraiser because the trust of neutrality of the assessment and dissatisfaction results in the disappointment of the appraisal system [1,14]. Again, the study identified some problems, which included failure to use PA for purposes of rewarding employees, career planning, possible promotion, giving feedback like encouraging employees who distinguish themselves better [15]. In 2006, Duffin *et al*. argued that healthcare appraisal system is inadequately executed in (GHS) [16]. Ohene-Saforo *et al*. [17] identified that the current GHS PAS does not meet the aspirations of the staff because it is characterized by certain flaws that ought to be addressed. Among the actual reasons for the failure of PAS was inadequate proper implementation due to the appraisers´ insufficient knowledge of the execution of the procedure [15]. Moreover, the outcome of the same study carried out by Worku *et al*. [15] indicated that the main problem with the PAS among healthcare workers was inappropriate implementation due to an absence of manager training [15]. Although, the fallouts from this study showed that there was agreement between managers and employees concerning the essence of PA, there were differences among them regarding the fairness of the scores, the resolution of the tools and the real use of the outcome of the assessment. The core mandate of every healthcare institution is to prevent, cure, or promote health in variety combinations. Whichever way the attention is, managing performance is key and critical activity that managers must carry out [10]. PA is a central responsibility of the healthcare system and improves employees´ morale and productivity [1,18]. This is because the performance of employees can be directly proportional to the performance of the organization.

The government of Ghana, through the Ministry of Health (MoH) and its agencies, recruit and post healthcare workers (HCW) across the nation. These HCW have specific job descriptions and are expected to be appraised as such. However, it is a public knowledge that most HCW only become serious about PA whenever their promotional interviews are approaching. Some employees in the GHS have developed different views and negative perceptions towards the service´s appraisal practice and its execution. Hence, describing it as a mere formality. The core issue of concern for most organizations and employees including GHS is whether PAS will truly be an adequate tool in maximizing employee´s performance. This could be attributed to the fact that most health facilities managers have no blueprints about PAS, especially in the area of continuous professional development and training. However, a major gap relates to the dearth of studies synthesizing the literature on PAS in Africa. The growing literature is scattered across different journals and publication outlets, with study settings usually limited to other continents and not Africa. This makes it extremely difficult to have a comprehensive and one-stop understanding of the development in the field of PAS. Similarly, it has hampered discussions on PAS in Africa, including prescribing areas where research may be needed most. Efforts to chart a pathway for a robust research agenda on PAS and as well as provide intuition into the directions and values of undertaking researches. In most Africa countries, especially Ghana, where most health facilities use PAS to evaluate their staff, there is paucity of literature regarding it. Even where such studies have been conducted, they were not carried out to assess the perceptions and practices. While it is prudent to explore the practices and perceptions of GHS staff regarding PAS, and to further unearth the perceived challenges for further investigations, as far as we know, and per our scholastic searches, none of such studies have been conducted in the Bono East Region (BER) of Ghana. Based on the aforementioned anecdotal evidence coupled with the unavailability of this study in the study setting, the researchers initiated this study to assess the practices and perceptions of the GHS employees on PAS in the Bono East Region of Ghana, West Africa.

## Methods

**Study setting:** the study was conducted in the Bono East Region. A new region, the Bono East Region, was created from the former Brong Ahafo Region. Techiman serves as the new region's capital. With a population of nearly one million people, Bono East has a land area of 22,952 square kilometers. The Bono East Region takes pride in being one of the states' regions with outstanding tourist attractions. The conservative character of the populace prevents industrial growth from contaminating their culture. With so many lovely tourist destinations, it becomes one of Ghana's most appealing cities. The Black Volta and River Tano, two of the most important rivers in the country and popular tourist destinations, pass through the area and are very beneficial to the local economy.

**Study design:** the study employed explorative institutional-based cross-sectional study. This study design was suitable because of the advantage of enabling researchers to compare several factors at once. Participants were recruited within a period of 16 weeks from August 2022 to November 2022.

**Study population:** the study included all HCWs within the BER who were in active service and had consented to freely participate in it. HCWs who were on retirement, those doing national service, and students on industrial/clinical attachments were not recruited to participate in the study. Those who met the inclusion criteria but were unwilling to participate were also excluded from the study.

**Sample size and sampling techniques:** the study's sample size was estimated using the Taro Yamane formula [19]:


$$n=\frac{N}{1+N{\left(e\right)}^{2}}$$

Where n denotes the sample size, N denotes the total target population, and e denotes the margin of error.


$$n=\frac{4,867}{1+4867{\left(0.05\right)}^{2}}=369.6$$

The total population of health care professionals in the Bono East Region is 4,867. Using a confidence level of 95%, 5% margin of error and 50% response distribution, the sample size was estimated to be 370. The sample size was then increased to 375 to take care of non-compliance and non-response rate. Thus, a total of 375 HCWs were recruited for this study.

The study utilized the simple random sampling method to recruit the study units. The list of all HCWs was obtained from the Regional Health Directorate, Human Resource Unit in a Microsoft Excel format. Using the randomization function of the Microsoft Excel software, the list was randomized and the first 50 names on the spreadsheet were considered. The process was repeated after every 50 responses to give equal chances to all health professionals in the region. Persons who were selected yet declined to participate in the study were replaced. The process was repeated until all the 375 sample units were exhausted.

**Data collection tools and procedure:** a questionnaire was used to gather the information. The questionnaire was adapted to match the objectives of the study and the study setting from earlier research [10,15,16]. The questionnaires were distributed to the participants for completion and return to the researchers because the majority of respondents could read and write in English. Section A of the questionnaire included questions on the socio-demographic characteristics of the respondents. Section B presented questions on respondents´ practices towards PA. Section C also presented questions on respondents´ perceptions and challenges relative to PA. The questionnaire was pretested among 15 HCWs in the Bono Region which shares a geological boundary with the study setting and has similar socio-economic and demographic characteristics as those in the study setting. This was to ensure the reliability and consistency of the instrument. The appropriate corrections were affected before the actual data collection was carried out on the study participants.

Following the consent form duly signed, a link was emailed to the HCWs' WhatsApp numbers so they could reply to the questionnaire. The electronic questionnaire was created such that it could not be submitted twice from the same device (a computer or mobile phone) to prevent such people from attempting the questionnaire more than once. Second, to attempt the questionnaire items, health professionals without WhatsApp numbers were called directly and questions were read out to them to provide their answers while the caller (one of the authors) recorded his/her response. Third, a hard copy questionnaire was also made available for those willing and requested for hard copies. Fourth, those who wished to answer the questionnaire through email also received a paper copy in the mail.

**Data analysis:** plans were made to guarantee that the data was input into Microsoft Excel in a way that would increase accuracy. All questionnaires were reviewed for completeness prior to data entry. After the formal data analysis was finished, the data were cleaned in Microsoft Excel and imported into Stata version 14. The dataset was normally distributed as evidenced by insignificant levels of the Kolmogorov-Smirnov test (p=0.145). The Kolmogorov-Smirnov test was used because the sample size was more than 50 [20]. Univariate and multivariate logistic regression was run to identify the determinants of performance appraisal completions. Tables and a figure were used to present the results. A p-value of less than 0.05 was deemed statistically significant.

**Ethical clearance:** the 1964 Declarations of Helsinki revised in 2013 were strictly followed in the conduct of the study [21]. First, the Committee on Human Research, Publications & Ethics' ethical approval was acquired (Reference Number: CHRPE/AP/765/22). All health facilities gave their approval for this project. Each respondent gave oral and written consent before taking part in the study. Sufficient information regarding the study´s goals was given to the participants. They were adequately assured of anonymity and confidentiality. Similarly, they were guaranteed of data safety and appropriate data usage and storage on the digitized questionnaire. The study's participants were made aware that taking part was completely voluntary and that they may withdraw at any time if they so choose. All participants were made aware that the general public will have access to the study's findings. Only participants who consented were allowed to participate in the study. All participants´ personal identifiers were deleted from the summarised data, ensuring confidentiality.

## Results

**Socio demographic characteristics of the respondents:** more than half (52.3%) of the respondents were within the ages 30 to 39 years with mean age of 33.9 ± 6.5. Majority of the respondents (58.4%) were males, 96.0% had tertiary education, 54.9% were Christians, 62.9% were clinical staff and 51.7% being married. A higher proportion of the respondents (40.3%) had 3 to 7 years of experience and 34.7% of the respondents had degree as their highest qualification (Table 1).

**Table 1:** socio-demographic characteristics of the respondents (n=375)

Variable	Categories	Frequency	Percentage
**Age**			
	20-29 years	105	28.0
	30-39 years	196	52.3
	40-49 years	63	16.8
	50 years and above	11	2.9
	Maximum		55 years
	Minimum		20 years
	Mean ± SD		33.9 ± 6.5
**Gender**			
	Female	156	41.6
	Male	219	58.4
**Level of education**			
	SHS	15	4.0
	Tertiary	360	96.0
**Qualification**			
	Certificate	77	20.5
	Diploma	121	32.3
	Degree	130	34.7
	Postgraduate	47	12.5
**Employment status**			
	Permanent	325	86.7
	Casual	50	13.3
**Religion**			
	Islam	139	37.1
	Christianity	206	54.9
	Traditionalist	30	8.0
**Marital status**			
	Married	194	51.7
	Single	111	29.6
	Co-habitation	54	14.4
	Separated/divorced	16	4.3
**Experience**			
	<3 years	89	23.7
	3-7 years	151	40.3
	>7 years	135	36.0
**Category of staff**			
	Non-clinical	139	37.1
	Clinical	236	62.9
**Average monthly income**			
	< GHS 2,000.00	227	60.5
	> GHS 2,000	148	39.5
	Minimum		GHS 300.00
	Maximum		GHS 8,700.00
	Mean ± SD		1946.4 ± 798.8

SHS: senior high school; GHS: Ghana health service

**Practices and perceptions of GHS employees about performance appraisal system (PAS):** the study revealed that majority of the respondents (86.7%) had completed performance appraisal (PA) form. Out of which 47.7% complete and submits PA every year, followed by those who complete and submit during promotion time (24.3%), every half year (15.1%) and anytime (12.9%). Over half of the GHS staff was trained on how to complete PA form and 52.8% were satisfied with how PAS are used to evaluate their performances. On perception of GHS employees towards PAS, majority of the respondents (54.4%) think the current PA is not fair, 60.5% indicated leaders take PA discussion seriously and nearly 60.0% believed PA actually measures their performance level. Table 2 shows the practice and perception of GHS employees about performance appraisal system.

**Table 2:** practice and perception of GHS employees about performance appraisal system (n=375)

Variables	Categories	Frequency	Percentage
**Ever completed PA form**			
	Yes	325	86.7
	No	50	13.3
**When do you complete and submit PA form (n=325)**			
	Every half-year	49	15.1
	Anytime	42	12.9
	Promotion time	79	24.3
	Yearly	155	47.7
**Received training to complete PA form**			
	Yes	201	53.6
	No	174	46.4
**Satisfied with how appraisals used to evaluate your performance**			
	Yes	198	52.8
	No	177	47.2
**Current PA fair**			
	Yes	204	54.4
	No	171	45.6
**Leaders take PA review discussions seriously**			
	Yes	227	60.5
	No	148	39.5
**Does PA tell your level of performance?**			
	Yes	223	59.5
	No	152	40.5

PA: performance appraisals

**Figure 1:**
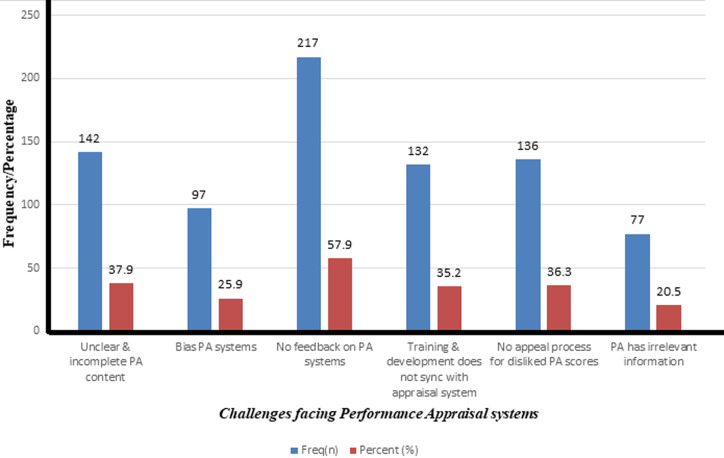
challenges facing PA systems among GHS employees (n=375)

**Challenges facing PA systems among GHS employees:** Figure 1 shows the barriers to PAS completions; 57.9% indicated they do not get feedback on the PA assessment, 36.3% mentioned that there are no appeal processes for disliked PA scores. Also, training and development does not sync with appraisal system (35.2%), unclear and incomplete PA content (37.9%), bias PA systems (25.9%) and PA has irrelevant information (20.5%) were other challenges identified by respondents.

**Factors influencing completion of performance appraisals systems among GHS employees:** Table 3 shows the factors influencing completion of performance appraisals systems among GHS employees. The study revealed that respondents who are 30 years and above are 2.24 times more likely to complete PAS as compare to those who are less than 30 years (AOR=2.24, 95% CI: 1.08-4.62). Also, males were more likely to complete PAS as compared to females among GHS employees (AOR=2.38, 95% CI: 1.16-4.91). Furthermore, GHS employees who were permanently employed are nearly 3.0 times more likely to complete PAS as compared to the non-permanent employees (AOR=2.9, 95% CI: 1.07-7.86).

**Table 3:** factors influencing completion of performance appraisals systems among GHS employees

		PP systems utilization						
Variable	Categories	Yes	No	p value	COR*	p value	AOR*	P value
**Age**				**p<0.001**				
	< 30 years	74(70.5%)	31(29.5%)		Ref*		Ref*	
	≥ 30 years	251(93.0%)	19(7.0%)		5.53(2.96-10.36)	p<0.001	2.24(1.08-4.62)	p=0.031
**Gender**				**p=0.056**				
	Female	129(82.7%)	27(17.3%)		Ref*		Ref*	
	Male	196(89.5%)	23(10.5%)		1.78(0.98-3.25)	p=0.058	2.38(1.16-4.91)	p=0.018
**Staff qualification**				**p=0.162**				
	Non degree	167(84.3%)	31(15.7%)		Ref*		Ref*	
	Degree	158(89.3%)	19(10.7%)		1.54(0.84-2.84)	p=0.164	1.36(0.61-3.09)	p=0.446
**Employment category**				**p<0.001**				
	Causal	33(66.0%)	17(34.0%)		Ref*		Ref*	
	Permanent	292(89.3%)	33(10.2%)		4.56(2.29-9.06)	p<0.001	2.90(1.07-7.86)	p=0.036
**Year of experience**				**p<0.001**				
	< 3 years	59(66.3%)	30(33.7%)		Ref*		Ref*	
	3-7 years	134(88.7%)	17(11.3%)		4.01(2.05-7.83)	p<0.001	5.53(2.48-12.36)	p<0.001
	> 7 years	132(97.8%)	3(2.2%)		22.37(6.57-76.22)	p<0.001	20.80(5.43-79.74)	p<0.001
**Category of staff**				**p=0.042**				
	Non-clinical	114(82.0%)	25(18.0%)		Ref*		Ref*	
	Clinical	211(89.4%)	25(10.6%)		1.85(1.02-3.37)	p=0.044	2.19(0.94-5.08)	p=0.068
**Average monthly income**				**p=0.246**				
	< GHS 2,000.00	193(85.0%)	34(15.0%)		Ref*		Ref*	
	> GHS 2,000	132(89.2%)	16(10.8%)		1.45(0.77-2.74)	p=0.248	0.55(0.233-1.30)	p=0.171

* AOR: adjusted odds ratios, COR: crude odds ratios, Ref: reference; GHS: Ghana health service; PP: performance appraisals

Finally, respondents who had worked for 3 to 7 years (AOR=5.53, 95% CI: 2.48-12.36) and those with over 7 years working experience (AOR=20.80, 95% CI: 5.43-79.74) were more likely to complete PAS as compared to their counters who worked less than 3 years.

## Discussion

Performance appraisals (PA) is the analysis of the success and failure of an employee and the examination of their sustainability for training and promotion future. PAS perceptions include if the PA provides positive or negative impact to the employees showing whether the employees get motivated to perform better; if they get a good feedback or do they get demotivated and lose interest in their jobs [13]. The current study assessed the practices and perceptions of the GHS employees on PAS in the Bono East Region of Ghana, West Africa. The survey found that although many respondents had completed PA, an encouraging proportion of them did not finish it within the anticipated time frame. Age, gender, mechanized employment, and length of employment (more than three years) were predictors of PAS.

Outcome from this study revealed that majority of the respondents had completed PA forms before but less than half of them do that every year. This is in line with 78.1% of reported to have completed PA in Ethiopia [22], 73.5% in Kenya [10], and 69.3% in Ghana [16]. These similarities could be that these studies were conducted in the same geological continents which have almost similar socio-economic demographic features. In contrast to the current findings, in Kashan University of Medical Sciences in the Northern Iran [8], and in Asian Istanbul [22], 44.5% and 39.5% of the respondents completed PA respectively. The incongruence in the findings may be likely due to the disparities in the sampling and the target populations. The former studies focused on banking and education while this study was on the health sector which might have determined the possibility of seriousness attached to PA especially when this area deals with human lives. Further studies are suggested to discover the determinants of job variations in identifying employees´ practices of PAS. Again, it is needful to re-evaluate the methodologies and scopes of training largely on PAS refresher courses in healthcare facilities and to adopt methods that would place premiums on the understandings of employees regarding PAS.

On the perceptions of respondents towards PAS, majority of them opined that the PA is not fair, most of respondents mentioned that their leaders consider PA discussions seriously and majority declared that PA assist them to evaluate their performances. The current findings are supported by similar studies [4,23] where they revealed that employees perceived that employees´ justice perceptions create a holistic comprehension within the minds of employee about quality of work life and the general working environment that suits their expectations in respect of fairness, morality and intrapreneurship then their attachment, satisfaction, motivation and performance increase. Additionally, these outcomes from this current study support previous studies [10,15]. It is our considered view that when employees are satisfied on the institutionalization of PA, they will perceive well and employ all the relevant possible efforts to adequately conduct their responsibilities and duties assigned them making their institutions more robust, productive and efficacious. In support of this, it has been argued publicly that, in order to determine employees behavior and future development, employees ought to experience positive reactions in the practice of PA else any PAS would be prone to possible failure [13]. It is instructive to note that the perceptions of procedural unfairness could adversely affect employees´ organizational commitment, job satisfaction, trust in management, performance as well as their work-related stress, organization citizenship behavior, thefts and inclination to litigate against their employer. Therefore, it is important for further studies to be carried out to establish the perceived determinants of employee´s perceptions about PA principally among healthcare professionals to unearth the factors that influence their perceptions on PAS and how that could largely inform their job descriptions. This may appraise policy makers and other key stakeholders on how to develop specific evidenced-based interventions that would reconsider employees´ perceptions on PAS to promote productivity in achieving the universal health coverage.

Insights from this study most importantly concerning the obstacles to PAS completion, most of the study participants mentioned they do not receive feedbacks on their PA assessments, no appeal processes for discontented PA score and training and development that do not synchronize with the PAS. This finding is in line with discoveries from another study conducted among Ghana Education Service (GES) employees in Bolgatanga Municipality of Ghana [24]. Although, the study participants are different in the studies, the concept of PAS is the same across all professions. One of the main challenges of PA arises if it becomes the primary tool for supervisors to give feedbacks. In the case of annual PAS, it surmises that employees spend 11 months of the year with no idea how they are progressing in terms of their performance. It is worthy to note that, in the spirit of providing feedback to staff development, PAS are supposed to be evaluated mid-year. Unfortunately, this is usually not the case as corroborated by the current finding where a higher proportion of the respondents completed their annual forms once in a year. As have been revealed in this study, some employees complete PAS retrospectively (i.e. when they are due for promotion) which defeats the essence of PAS. Frequent feedback by managers to employees is the major solution to the challenge of employees not receiving feedbacks on their PA. Although they may not appreciate critical feedbacks they would not be blindsided if they get that on regular basis. This could be done face-to-face or through electronic mail. During the period of PA, employees would answer better if the feedback is based on how they perform compared with a year or two years ago, as opposed to how they compare with their coworkers in the present. Most employees are of the view that measuring against themselves is much fairer and more objective than colleague comparisons, so they may take it more seriously. There should be frantic modalities in place to ascertain how employees prefer receiving their feedbacks from PA and there should be policy engagements on how to restructure the components of PA, their trainings and development to synchronize PAS in Ghana.

Regarding the determinants of PAS completion, it was revealed that increasing age was an influencing factor to the completion of PA. The inference here could be that the more employees grow in a profession the more experience they gain and become vigilant about a lot of protocols and formalities. It could be that, as they mature in the profession, they decide to be serious about their performance which may come with consequential benefits like salary increments hence completing their PA.

The study further revealed that males were more likely to complete PAS as compared to females. Whilst it is difficult to find same expression of this related studies, Szameitat *et al*. [25] argued that women are better in multitasking compared to men as a result of juggling work, family, and/or children. By extension, they expect women to be able to cope with work and its attendant responsibilities including completion of PAS. By this argument, the temptation to say women might have been occupied with domestic activities therefore would not be enough justification for more men completing PAS than females. According to Ring *et al*. [26] women are often less competitive and self-assured than males. Thus, males will be active in seeking feedback in PAS so as to enable them make advancement in their carrier. Whilst it is very difficult to get a direct justification for this relationship, it provides the best explanations to males completing PAS than females.

Also, respondents who were employed permanently were noticed to be determinants to the completion of PA. As mechanized staff, their activities are regulated and supervised by their employers and immediate bosses with blueprints unlike non-mechanized staff who are employed and supervised by the facility managers. The former has job securities with laid down promotional structures that require PA, hence, a determinant to the completion of PA.

In relation to the above, years of work in the service was seen as a considerable factor influencing the completion of PA. The attrition could be that as employees stay and work longer, they get used to the norms and practices of the institution and go strictly to them with ease. Efforts should be made to delve into these determinants to ascertain their veracities to ensure that adequate scientific trainings are geared towards that to encapsulate the majority group who do not fall in this age bracket, cadre of staff and years or level of working experience to get a lot more employees completing the PA as required.

**Limitation:** there is insufficient literature on the topic in Ghana, particularly in the GHS, hence, the difficulty in using the appropriately linked literature to validate or refute the study outcomes. Similarly, because human beings are tending to hide whatever they actually feel from within due to different reasons, respondents might be hesitant to produce their real feelings as a result conclusions of the findings might be affected. To deal with this, respondents were assured that their response will be anonymous and encouraged to be real as much as possible. Lastly, as with all cross-sectional studies, cause-effect relationship is not intended. Although, our sample size is representative to the study area, the generalization of our findings to Ghana should be done with extreme caution.

## Conclusion

The study identified that a significant number of the respondents even though completed PA, quite an encouraging number of them do not complete PA at the required period as expected. Increasing age, gender, mechanized job and working experience more than 3 years were significant determinants to completion of PA form. Continuous professional development for GHS staff on PAS is required. It is further recommended that the MoH in collaboration with the GHS human resource division should organize stakeholder´s engagement with facilities managers and employees on the PAS in all the departments to inform all managers and employees on what is required from everyone. Managers ought to provide timeous feedbacks on PA to aid employees to know exactly where they need to improve, and the appraisers must be given the requisite training to sharpen their assessment skills.

### What is known about this topic

- *Performance appraisal is a recognized practice in institutions that is grounded in objective responsibilities with mental components, compares performance and project performance;*
- *During the World War II era, performance appraisal systems was established in the United States of America (USA) in the 1940s;*
- *It served as a means of accurately determining employees' compensation at the time, which was referred to as merit rating*.

### What this study adds

- *The study identified that majority of the respondents completed PA, but quite an encouraging number of them do not complete PA at the required period as expected;*
- *Age, male staff, permanent employment and increasing years of experience were the significant predictors of PA completion;*
- *The study recommends that the GHS human resource division should organize stakeholder´s engagement with facilities managers and employees on the PAS in all the departments to inform all managers and employees on what is required from everyone*.

## References

[1] Moradi T, Mehraban MA, Moeini M (2017). Comparison of the Perceptions of Managers and Nursing Staff Toward Performance Appraisal. Iran J Nurs Midwifery Res.

[2] Lawler III EE, Benson GS, McDermott M (2012). What makes performance appraisals effective?. Compens Benefits Rev.

[3] Awases MH, Bezuidenhout MC, Roos JH (2013). Factors affecting the performance of professional nurses in Namibia. Curationis.

[4] Roine A (2018). Employee perceptions of performance appraisal.

[5] Lim CT, Ahmad N (2021). The relationship between human resource management practices and employee performance. Res Manag Technol Bus.

[6] Canet-Giner T, Redondo-Cano A, Saorín-Iborra C, Escribá-Carda N (2020). Impact of the perception of performance appraisal practices on individual innovative behavior. Eur J Manag Bus Econ.

[7] Cappelli P, Conyon MJ (2018). What do performance appraisals do?. ILR Rev.

[8] Golafshani SH, Ansaritabar A, Hoseindoost G, Atharizadeh M (2018). Attitude of staff of Kashan university of medical sciences concerning their annual performance. Int Arch Heal Sci.

[9] Sepahvand F, Mohammadipour F, Parvizy S, Zagheri Tafreshi M, Skerrett V, Atashzadeh-Shoorideh F (2020). Improving nurses´ organizational commitment by participating in their performance appraisal process. J Nurs Manag.

[10] Kwamifoli DK (2017). Perceptions of fairness of performance appraisal and organizational commitment among employees of Ghana health service in Cape Coast and Ho. University of Cape Coast.

[11] Choudhary GB, Puranik S (2014). A study on employee performance appraisal in health care. Asian J Manag Sci.

[12] Majidi S, Daneshkohan A, Zarei E, Ashktorab T (2021). Perspectives of health workers on annual performance appraisal: A study in primary health care. Int J Healthc Manag.

[13] Bekele AZ, Shigutu AD, Tensay AT (2014). The effect of employees´ perception of performance appraisal on their work outcomes. Int J Manag Commer Innov.

[14] Murphy KR (2020). Performance evaluation will not die, but it should. Hum Resour Manag J.

[15] Worku Z (2019). A Study Of Employee Perceptions About Performance Appraisal At Transnet Engineering, South Africa. J Appl Bus Res.

[16] Duffin C (2006). How should the government check nurses are fit to practise? The Department of Health has set out ideas to replace the PREP system of revalidation. It wants your views. Nurs Stand.

[17] Ohene-Saforo EVA (2021). Employees' Perception of Appraisal and Job Performance in Ghana Health Service.

[18] Lee F-H, Lee T-Z, Wu W-Y (2010). The relationship between human resource management practices, business strategy and firm performance: evidence from steel industry in Taiwan. Int J Hum Resour Manag.

[19] Yamane T (1967). Elementary sampling theory. Prentice-Hall. Inc., Englewood Cliffs.

[20] Mishra P, Pandey CM, Singh U, Gupta A, Sahu C, Keshri A (2019). Descriptive statistics and normality tests for statistical data. Ann Card Anaesth.

[21] World Medical Association (2013). Declaration of Helsinki, Ethical Principles for Scientific Requirements and Research Protocols. Bull World Health Organ.

[22] Birecikli B, Alpkan L, Ertürk A, Aksoy S (2016). Employees ´ need for independence, organizational commitment, and turnover intentions: The moderating role of justice perceptions about performance appraisals. International Journal of Organizational Leadership.

[23] Ullah Z, Ahmad N, Scholz M, Ahmed B, Ahmad I, Usman M (2021). Perceived Accuracy of Electronic Performance Appraisal Systems: The Case of a Non-for-Profit Organization from an Emerging Economy. Sustainability.

[24] Amilariba AD (2021). Performance Appraisal and Employee Performance in Ghana Education Service in Bolgatanga Municipality. University of Cape Coast.

[25] Szameitat AJ, Hamaida Y, Tulley RS, Saylik R, Otermans PCJ (2015). “Women are better than men”-Public beliefs on gender differences and other aspects in multitasking. PLoS One.

[26] Ring P, Neyse L, David-Barett T, Schmidt U (2016). Gender Differences in Performance Predictions: Evidence from the Cognitive Reflection Test. Front Psychol.

